# Geographic variation in point of care ultrasound provision: results from a national audit

**DOI:** 10.1186/s13089-023-00314-9

**Published:** 2023-03-21

**Authors:** Sunil Aggarwal, Reshma Shanmugam, Thomas Knight, Catherine Atkin, Sarbjit Clare, Nicholas Smallwood, Daniel Lasserson

**Affiliations:** 1 University Hospitals Birmingham NHS Foundation Trust, Birmingham, UK; 2 Sandwell and West Birmingham Hospitals NHS Foundation Trust, Birmingham, UK; 3 Birmingham Acute Care Research Group, Institute of Inflammation and Ageing, University of Birmingham, Birmingham, UK; 4 Hampshire Hospitals NHS Foundation Trust, Hampshire, UK; 5 School of Medicine, University of Warwick, Coventry, UK; 6 Oxford University Hospitals NHS Foundation Trust, Oxford, UK

**Keywords:** Point-of-care systems, Ultrasonography, Acute medicine, Training

## Abstract

**Background:**

There is an emerging consensus that point-of-care ultrasound is an essential skill in acute care. This is reflected in recent changes to the Acute Internal Medicine curriculum in the UK. The need to develop and maintain specific ultrasound competencies is now a mandatory component of training. There is a degree of uncertainty as to how existing training infrastructure can best accommodate these changes.

**Methods:**

Data were obtained from the latest annual Society for Acute Medicine Benchmarking Audit 2021. All Acute Medical Units in the UK are eligible to participate. Data pertaining to the number of ultrasound machines and number of clinicians that regularly use point of care ultrasound were collected. This was used to develop a series of maps demonstrating variation in provision at the national level.

**Results:**

In total, 123 AMUs responded to the questions related to ultrasound prevalence and numbers of trained clinicians. Of these, 78.9% (97/123) reported having access to at least one ultrasound machine. There was at least one clinician that regularly used ultrasound in 81 responding hospitals (65.9%). There was significant geographic heterogeneity in the use of ultrasound and availability of accredited supervisors. At a regional level, ultrasound expertise is typically concentrated within a relatively small number of hospitals.

**Conclusion:**

Geographic variation in the use of ultrasound and availability of registered supervisors represents a significant challenge to ultrasound training provision at the national level. Targeted interventions in areas with less developed training infrastructure, such as regional training hubs may be required to ensure more equitable access to training opportunities.

## Introduction

Point-of-care ultrasound (POCUS) is increasingly advocated as a tool to optimise clinical decision-making in the acute setting. In contrast to departmental ultrasound performed by radiologists and ultrasonographers, POCUS involves a limited examination designed to answer well defined clinical questions. The use of ultrasound to enhance the safety of invasive medical procedures is well established, but its potential applications in acute care are far wider [1].

The Acute Medical Unit (AMU) is a defining feature of the acute care pathway in the UK. AMUs are a “dedicated facility within the hospital that acts as the focus for acute medical care for patients who have presented as a medical emergency to hospital” [2]. Delivery of care on these units is typically delivered by Acute Internal Medicine (AIM) specialists. AIM is a hospital specialty concerned with the assessment, diagnosis and treatment of adult patients with urgent medical needs [3, 4]. The ability to perform specific POCUS examinations is increasingly recognised as an essential skill in acute care. This is reflected in recent changes to the AIM curriculum.

Historically, POCUS has been viewed as a specialist skill within AIM developed at the discretion of the individual clinician. The updated AIM curriculum includes the acquisition of specific POCUS competencies as a core component of AIM training [5]. This represents a paradigmatic shift in approach, with the ability to undertake POCUS no longer viewed as an optional extra, but a prerequisite for completion of training in AIM and joining the specialist register. Incumbent on this change in approach is the need for AIM training programmes to provide the teaching and training resources needed to facilitate the acquisition of this additional skill.

The Society for Acute Medicine (SAM) endorses the Focused Acute Medicine Ultrasound (FAMUS) accreditation to demonstrate POCUS competency [6]. Successful completion requires attendance at a 1-day course followed by a period of supervised practice typically over a 6 to 12 month period. FAMUS accreditation is estimated to require a minimum of 22 h of direct and indirect supervision. A key obstacle to increasing the number of FAMUS-accredited clinicians is the availability of supervisors [7]. The time commitment of supervisors to undertake this essential role is not commonly recognised within job planning and is often provided informally.

Variation in the provision of ultrasound equipment and the number of clinicians with training in POCUS has previously been reported [8]. The change in curricular design will inevitably drive increased demand for training. The ability for existing training infrastructure to accommodate this increase in demand in the short term is unclear. We used the Society of Acute Medicine Benchmarking Audit (SAMBA) to better characterize geographical variation in POCUS provision to inform the design of future training.

## Methods

SAMBA is an annual audit and benchmarking exercise undertaken by the Society for Acute Medicine (SAM). Recruitment to SAMBA is open to all hospitals in the UK receiving acutely unwell (non-elective, adult) medical patients. Non-acute and community hospitals were excluded from participating. SAMBA consists of two separate components designed to collect information at the hospital and patient level. The SAMBA21 study protocol is publicly available [9] and the national report is published elsewhere [10]. Patient level data collection uses a single day of care methodology to collect information on performance against specific audit standards. Prior to the collection of patient level data, a clinician at each participating hospital completed an online survey to provide information relating to organizational structure and resource availability. The hospital-level survey asked “Do you have an ultrasound machine on your AMU?” and “How many non-training grade clinicians regularly use point of care ultrasound or echocardiography?” The responses were used to develop a better understanding of the geographical variation in POCUS provision in the UK. As AIM training is rotational, only counting non-training grade clinicians was felt more likely to provide a realistic measure of the hospitals capability to provide POCUS training over a sustained period. Details in relation to the location and number of FAMUS supervisors were publicly available on the FAMUS website. The relevant information was accessed on 1st July 2022.

### Maps

Maps were created using R version 3.5.3 (2019) (Copyright The R Foundation for Statistical Computing). Base maps of the UK were obtained from the Office of National Statistics with regional boundaries drawn at the International Territory Level 2 (ITL2). There are 47 ITL2 regional boundaries in the UK. Post graduate medical education in England is structured around 13 Local Education and Training Boards. The devolved nations have similar institutions with national coverage. The use of ITL2 level boundaries allowed geographical variation to be explored at a greater resolution than using boundaries defined by the much larger areas covered by Local Education Training Boards (LETBs) and their counterparts in the devolved nations. Both ITL2 boundaries and LETBs are used as a proxy to represent regional deaneries. Hospital location was mapped to geographic coordinates using the PostcodesioR package and rendered using the jeojsonio package. Publicly available data sources were used to obtain the locations of all hospitals with a Type 1 Emergency Department (major department providing consultant-led-24-h service) used as a proxy for hospitals eligible to participate in SAMBA. In all maps, the region of Greater London is depicted adjacent to the UK to allow for easier interpretation.

### Statistics

Descriptive data are presented as mean and standard deviation (SD) for normally distributed values and median and range for non-normally distributed values. Differences in the number of hospital and AMU beds between those with and without ultrasound were statistically compared using *t*-tests.

## Results

**Fig. 1:**
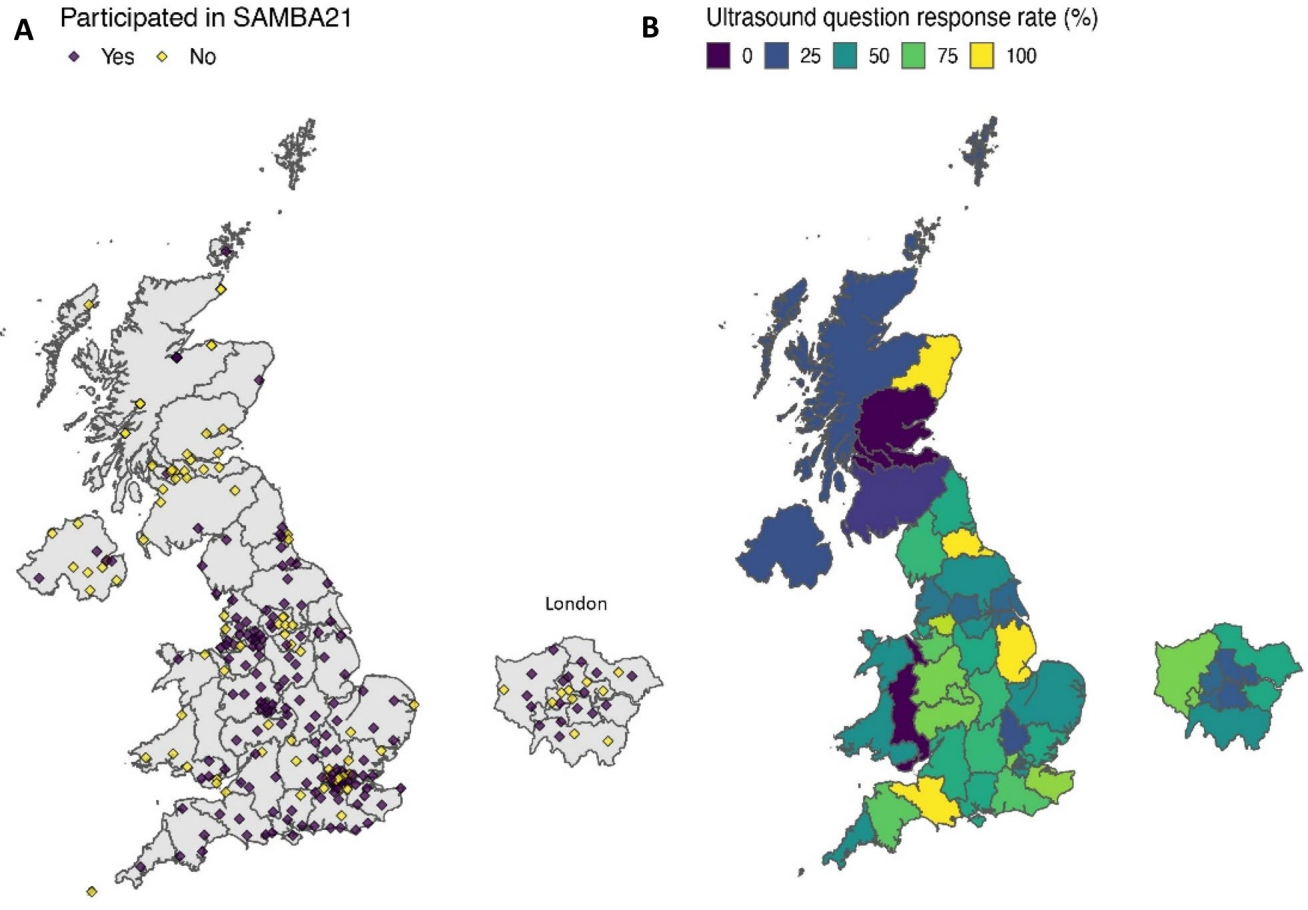
Map demonstrating coverage of SAMBA21. **A** SAMBA21 participating hospitals and non-participating hospitals. **B** Ultrasound question response rate at the regional level with total number of hospitals (SAMBA participating and non-participating) as the denominator *grey = no response to question

SAMBA21 collected data on organisational structure and resource availability from 153 hospitals. The response rate was 66.7% across all UK hospitals. This included 139 hospitals from England (response rate 79%), 5 hospitals from Scotland (response rate 17.2%), 4 hospitals from Northern Ireland (response rate 33.3%) and 5 hospitals from Wales (response rate 38.5%). Responses to the questions designed to ascertain the prevalence of ultrasound equipment and trained clinicians were provided by 123 hospitals (response rate 80.4%). A map demonstrating the location of SAMBA participating hospitals and regional variation in response rate in relation to the ultrasound specific questions is provided in Fig. 1.

**Fig. 2:**
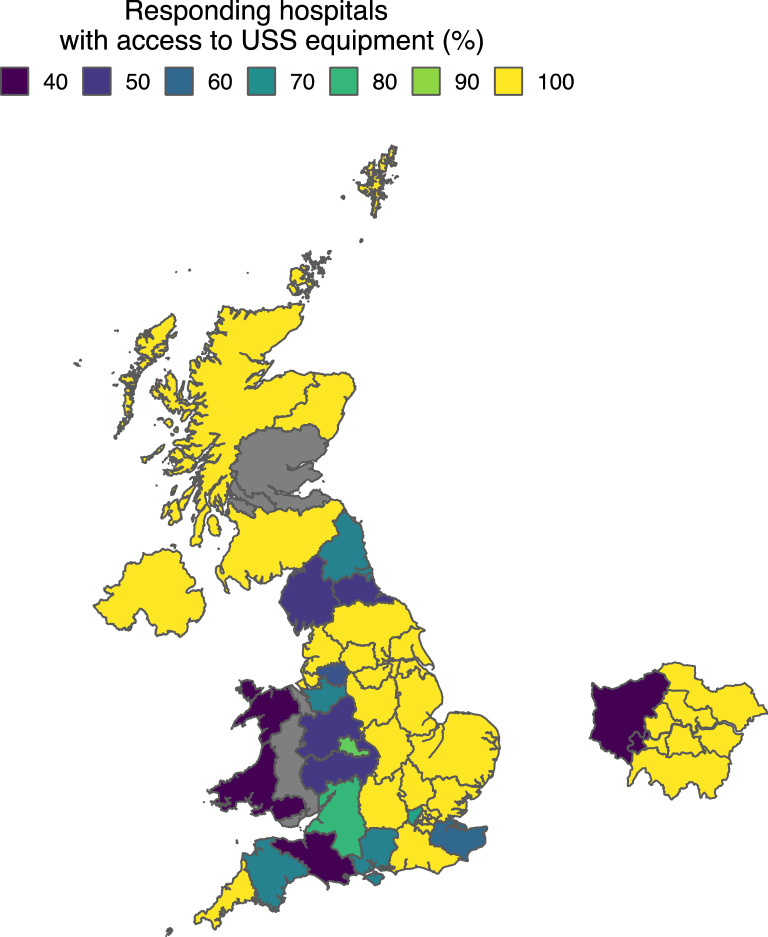
Geographical variation in access to ultrasound equipment at the regional level amongst SAMBA participating hospitals. *grey = zero responses available to calculate %

Access to dedicated ultrasound equipment on the AMU was reported in 97 (78.9%) hospitals. AMUs with access to dedicated ultrasound equipment tended to have more beds (with: mean 44 beds, without: mean 37 beds, *p*-value < 0.05) and were located within hospitals with a larger number of total beds (with: mean 582 beds, without: mean 456 beds, *p*-value < 0.05). At the regional level, the proportion of responding hospitals with direct access to US equipment on the AMU ranged from 40 to 100%. Geographical variation in access to equipment is provided in Fig. 2 and Table 1.

**Table 1:** Numbers of US machines, non-training grade POCUS users and FAMUS supervisors corresponding to each ITL2 territory (LETB deaneries shown in bold)

Region (ITL2 regional boundaries *n* = 37)	ITL2 regional boundary	Number of US machines (*n*)	Number of non-training grade POCUS users (*n*)	FAMUS supervisors (*n*)
**East Midlands**		***N* = 6**	***N* = 25**	***N* = 4**
Derbyshire and Nottinghamshire	TLF1	2	13	2
Lincolnshire	TLF3	2	4	0
Leicestershire, Rutland and Northamptonshire	TLF2	2	8	2
**East of England**		***n* = 8**	***N* = 24**	***n* = 9**
Leicestershire, Rutland and Northamptonshire	TLF2	0	0	1
East Anglia	TLH1	3	9	4
Suffolk	TLH14	1	0	1
Essex	TLH3	3	12	3
Bedfordshire and Hertfordshire	TLH2	1	3	0
**Kent Surrey Sussex**		***N* = 9**	***N* = 35**	***n* = 19**
Berkshire, Buckinghamshire and Oxfordshire	TLJ1	0	0	1
Surrey, East and West Sussex	TLJ2	6	26	16
Kent	TLJ4	3	9	2
**London**		***N* = 16**	***N* = 33**	***n* = 9**
Inner London—West	TLI3	2	3	2
Inner London—East	TLI4	2	7	3
Outer London—West and North West	TLI7	3	5	1
Outer London—East and North East	TLI5	3	9	0
Outer London—South	TLI6	3	4	0
Berkshire, Buckinghamshire and Oxfordshire	TLJ1	3	5	3
**North East**		***N* = 6**	***N* = 12**	***n* = 7**
Tees Valley and Durham	TLC1	2	4	3
Cumbria	TLD1	1	4	0
Northumberland and Tyne and Wear	TLC2	3	4	4
**North West**		***N* = 13**	***N* = 30**	***n* = 12**
Greater Manchester	TLD3	5	8	3
Lancashire	TLD4	3	4	2
Cheshire	TLD6	1	6	1
Merseyside	TLD7	4	12	6
**Northern Ireland**		***N* = 3**	***N* = 6**	***N* = 2**
Northern Ireland	TLN0	3	6	2
**Peninsula**		***N* = 3**	***N* = 2**	***n* = 4**
Cornwall and Isles of Scilly	TLK3	1	0	1
Devon	TLK4	2	2	3
**Scotland**		***N* = 4**	***N* = 13**	***n* = 13**
Highlands and Islands	TLM6	1	5	1
North Eastern Scotland	TLM5	1	6	5
Eastern Scotland	TLM7	0	0	1
West Central Scotland	TLM8	1	0	5
Southern Scotland	TLM9	1	2	1
**Severn**		***N* = 5**	***N* = 29**	***n* = 2**
Gloucestershire, Wiltshire and Bristol/Bath area	TLK1	4	27	1
Dorset and Somerset	TLK2	1	2	0
Cornwall and Isles of Scilly	TLK3	0	0	1
**Thames Valley**		***N* = 2**	***N* = 4**	***n* = 8**
Berkshire, Buckinghamshire and Oxfordshire	TLJ1	2	4	8
**Wales**		***N* = 2**	***N* = 6**	**Wales (*n* = 1)**
Berkshire, Buckinghamshire and Oxfordshire	TLJ1	0	0	1
West Wales and The Valleys	TLL1	2	6	0
**Wessex**		***N* = 3**	***N* = 14**	***n* = 4**
Hampshire and Isle of Wight	TLJ3	2	9	4
Dorset and Somerset	TLK2	1	5	0
**West Midlands**		***N* = 10**	***N* = 25**	**(*n* = 9)**
Herefordshire, Worcestershire, and Warwickshire	TLG1	2	4	0
Shropshire and Staffordshire	TLG2	2	4	3
West Midlands	TLG3	6	17	6
**Yorkshire**		***N* = 7**	***N* = 13**	***n* = 18**
East Yorkshire and Northern Lincolnshire	TLE1	1	0	1
North Yorkshire	TLE2	1	6	0
South Yorkshire	TLE3	2	3	12
West Yorkshire	TLE4	3	4	4
Derbyshire and Nottinghamshire	TLF1	0	0	1
Total		97	271	121

**Fig. 3:**
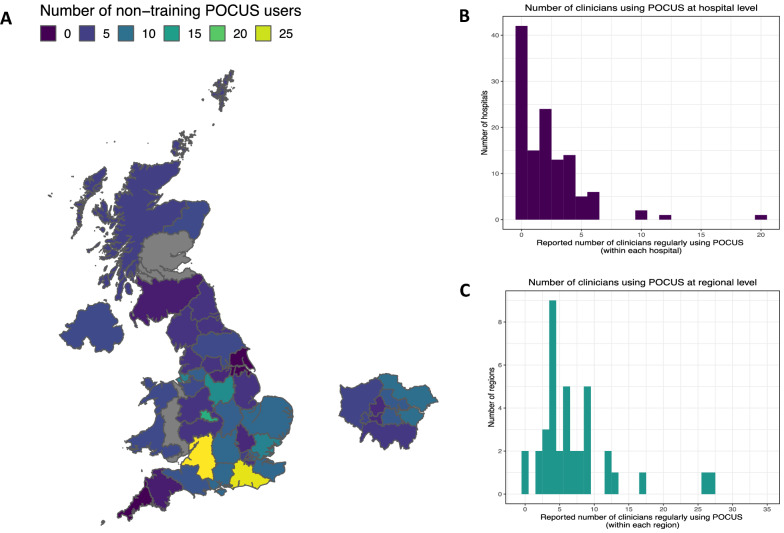
Geographical variation in the number of non-training grade clinicians that regularly use point of care ultrasound. **A** Map of demonstrating the number of clinicians within ITL2 boundaries. **B** Histogram showing counts at the Hospital level **C** Histogram showing counts at the ITL2 level. *grey = no response to question

There was variation in utilization of POCUS by non-training grade clinicians at the national level. A total of 271 clinicians were reported to regularly use POCUS located across 81 hospitals. The median number of clinicians that regularly used POCUS per hospital was 2 (range 0–20). POCUS was not regularly utilized by any non-training grade clinicians in 42 (34.1%) hospitals. There was considerable variation in POCUS use at the regional level (Table 1, Fig. 3). Geographical differences at a regional level were largely driven by a small number of hospitals with a relatively large number of clinicians that regularly utilized POCUS (Fig. 3).

**Fig. 4:**
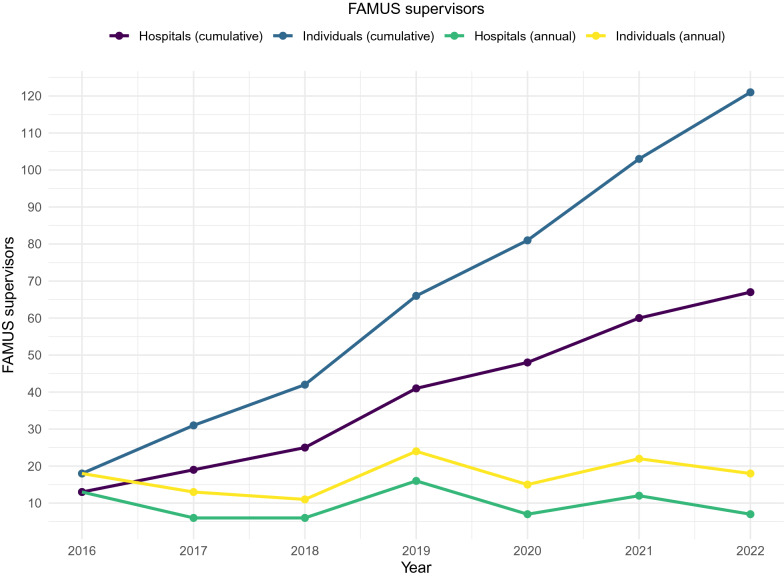
Annual and cumulative growth rate of FAMUS supervisors aggregated at the national level and hospital level

There are currently 121 registered FAMUS supervisors. The average incidence of FAMUS registered supervisors is 17.1 (SD 4.6) per year (Fig. 4). At the post graduate deanery level, all jurisdictions had at least one FAMUS supervisor (median 8, range 1–19) (Table 1). At a regional level, FAMUS supervisors were present in 42 (89.4%) regions (median 3, range 0–16). At least one FAMUS supervisor was present in 67 hospitals (29.1%). In hospitals with at least one FAMUS supervisor, the median number of supervisors was 1 (range 1–6).

**Fig. 5:**
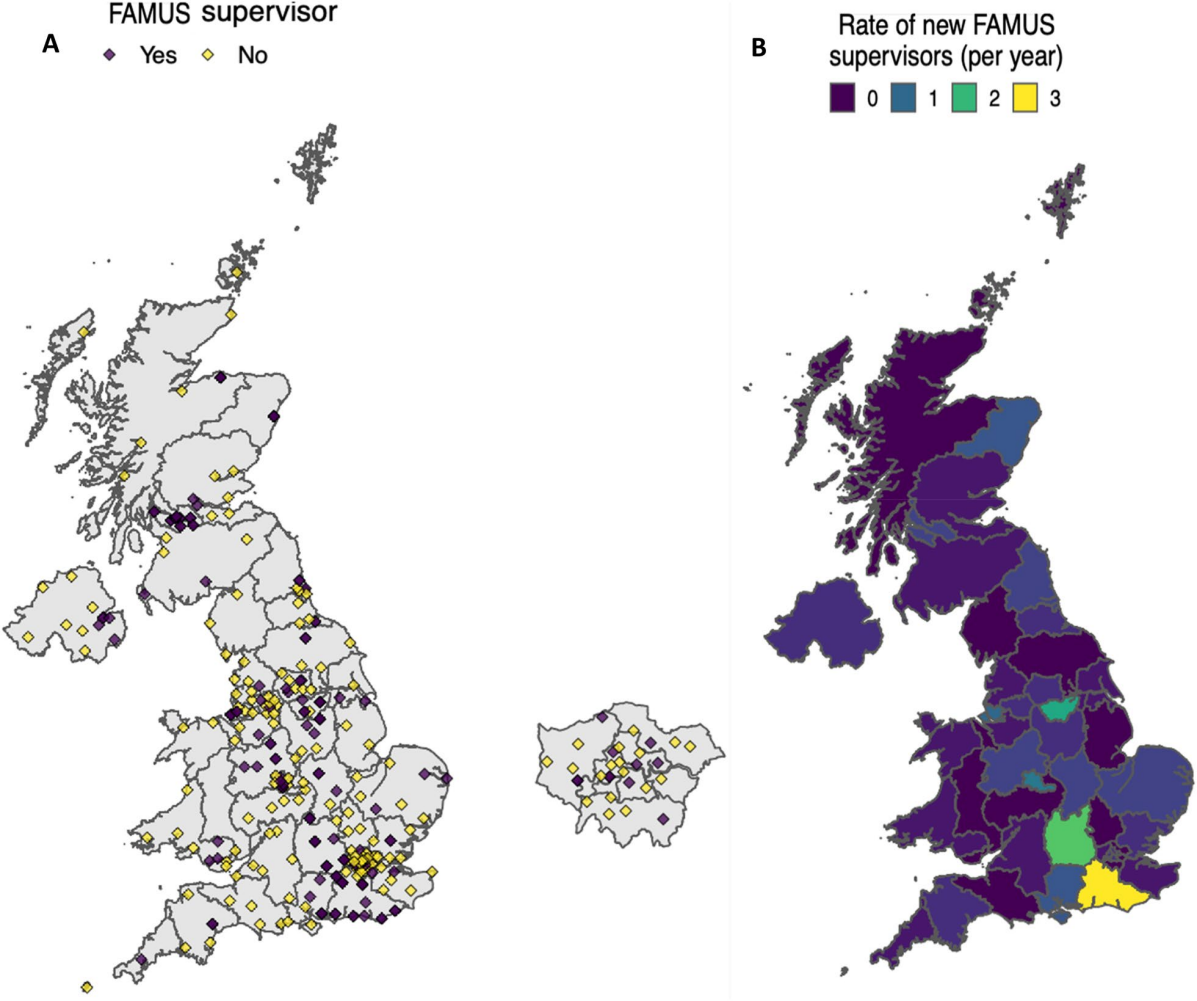
Geographical distribution of FAMUS supervisors in the UK. **A** All hospitals in the UK with a type 1 ED stratified by the presence of at least one FAMUS supervisors. **B** Regional variation in the growth rate of FAMUS supervisors

New FAMUS registered supervisors were more likely to emerge from hospitals with an existing FAMUS supervisor. The average number of FAMUS supervisors registering in hospitals without a prior FAMUS supervisor is 9.5 (SD 4.1) per year. Regional differences in the presence of FAMUS supervisors are demonstrated in Fig. 5. The national growth rate in the number of FAMUS supervisors was largely driven by increases in a small number of geographical regions (Fig. 5). The average rate of growth was greater than 1 FAMUS supervisor per year in 4 (8.5%) regions.

## Discussion

The ability to perform POCUS is increasingly recognized as an essential skill in acute care. The new AIM curriculum places a clear emphasis on the acquisition of POCUS competency and will be a powerful catalyst in upskilling the medical workforce. Prioritization of POCUS in the curriculum must be accompanied by similar expansion of training infrastructure. There is currently significant variation in the availability of ultrasound equipment, the utilization of POCUS by clinicians and the availability of FAMUS supervisors at the regional level. This is likely to create inequality in access to training opportunities that must be addressed to ensure the trainees are able to meet the requirements of the new curriculum.

Most hospitals participating in SAMBA21 reported access to dedicated ultrasound equipment. The prevalence of ultrasound equipment on the AMU was 78.9%. This represents an increase in prevalence from 58.1% reported in SAMBA19 [8]. Regional variation in access to dedicated ultrasound equipment represents an important consideration from the perspective of national training. Given the rotational nature of AIM training it is important that all hospitals have ready access to equipment to ensure competencies can be both acquired and maintained. The provision of portable ultrasound machines on all AMUs is likely to require substantial financial investment. An alternative solution would be to provide all clinicians in training with an individual hand-held ultrasound device. This approach may have both practical and cost advantages relative to investment in traditional portable machines [11].

There is a large differential between the number of clinicians that regularly use POCUS and the number of registered FAMUS supervisors. This may reflect the presence of a pool of clinicians with the ability to provide supervision and mentorship that are not currently formerly engaged with the FAMUS process. These clinicians may have accredited through an alternative process or may have acquired competencies prior to the existence of a formalized accreditation process. Additionally, some doctors may be formally accredited with FAMUS but not registered as supervisors. The possibility of clinicians regularly utilizing ultrasound without prior training or accreditation cannot be excluded from the information collected within our evaluation and could represent a significant governance risk.

Clinicians that regularly utilize POCUS appear to be concentrated in specific hospitals and in specific geographic regions. This is likely to be a consequence of the previous training model, driven primarily by the enthusiasm of early adopters in the absence of a more systematic approach. The pattern is mirrored in the distribution of FAMUS supervisors. A consistent annual increase in the number of individuals registered as FAMUS supervisors is an encouraging sign, but this increase is not uniform across the system. Much of the observed growth has occurred in hospitals with existing expertise and the growth rate of hospitals without a registered FAMUS supervisor is considerably slower.

The concentration of expertise in a limited number of locations represents a significant challenge to training provision. Improving access to training opportunities will require an increase in both the absolute number of supervisors and an increase in the number of hospitals able to provide training. The development of regional POCUS training hubs accompanied by enhanced use of remote supervision offers a potentially viable means to address this issue. The use of cloud-based platforms able to stores anonymized images could enable supervision to be extended to hospitals without an established local training program and allow supervisory periods to be stretched beyond the time limits imposed by rotational training [12]. This would require collaboration between hospitals to provide a robust governance structure and ensure safe oversight. It is imperative that the significant time burden associated with the development and delivery of POCUS training programs is formally recognized within job planning.

## Limitations

SAMBA provides unique insights at the system level. Not all eligible hospitals participate in SAMBA and there may be systematic differences between SAMBA-participating and non-SAMBA participating hospitals which affect generalizability. Geographic variation should be interpreted in the context of differences in SAMBA participation rates and the response rate to specific ultrasound questions. The response rate was lower than average in Scotland and in London so conclusions may be unreliable in these areas. The regional boundaries used to create the maps were based on OSN intranational territory boundaries rather than boundaries that define hospitals within the same post-graduate organization.

## Conclusion

There remains variability in the provision of ultrasound resources, the number of clinicians that utilize POCUS and the number of supervisors between different regions of the country. These disparities will need to be addressed for national changes in the AIM curriculum to be successfully accommodated.

## Data Availability

The data that support the findings of this study are available from the Society for Acute Medicine but restrictions apply to the availability of these data, which were used under license for the current study, and so are not publicly available. Data are, however, available from the authors upon reasonable request and with permission of the Society for Acute Medicine.

## Abbreviations

AIM: Acute Internal Medicine
AMU: Acute Medical Unit
FAMUS: Focused Acute Medicine Ultrasound
ITL2: International Territory Level 2
LETB: Local Education and Training Boards
OSN: Ordinance Survey National
POCUS: Point of care ultrasound
SAM: Society for Acute Medicine
SAMBA: Society of Acute Medicine Benchmarking Audit
SD: Standard deviation
UK: United Kingdom
US: Ultrasound
